# Maternal Glycaemic and Insulinemic Status and Newborn DNA Methylation: Findings in Women With Overweight and Obesity

**DOI:** 10.1210/clinem/dgac553

**Published:** 2022-09-22

**Authors:** Marion Lecorguillé, Fionnuala M McAuliffe, Patrick J Twomey, Karien Viljoen, John Mehegan, Cecily C Kelleher, Matthew Suderman, Catherine M Phillips

**Affiliations:** School of Public Health, Physiotherapy and Sports Science, University College Dublin, Dublin 4, Republic of Ireland; UCD Perinatal Research Centre, School of Medicine, National Maternity Hospital, University College Dublin, Dublin, Ireland; School of Medicine, University College Dublin, Dublin, Republic of Ireland; School of Public Health, Physiotherapy and Sports Science, University College Dublin, Dublin 4, Republic of Ireland; School of Public Health, Physiotherapy and Sports Science, University College Dublin, Dublin 4, Republic of Ireland; School of Public Health, Physiotherapy and Sports Science, University College Dublin, Dublin 4, Republic of Ireland; MRC Integrative Epidemiology Unit, Population Health Sciences, Bristol Medical School, University of Bristol, Bristol, UK; School of Public Health, Physiotherapy and Sports Science, University College Dublin, Dublin 4, Republic of Ireland

**Keywords:** lifestyle intervention, overweight and obese pregnancy, maternal dietary glycemic indices, maternal glycaemia, insulin concentrations, DNA methylation

## Abstract

**Context:**

Maternal dysglycaemia and prepregnancy obesity are associated with adverse offspring outcomes. Epigenetic mechanisms such as DNA methylation (DNAm) could contribute.

**Objective:**

To examine relationships between maternal glycaemia, insulinemic status, and dietary glycemic indices during pregnancy and an antenatal behavioral-lifestyle intervention with newborn DNAm.

**Methods:**

We investigated 172 women from a randomized controlled trial of a lifestyle intervention in pregnant women who were overweight or obese. Fasting glucose and insulin concentrations and derived indices of insulin resistance (HOMA-IR), β-cell function (HOMA-%B), and insulin sensitivity were determined at baseline (15) and 28 weeks’ gestation. Dietary glycemic load (GL) and index (GI) were calculated from 3-day food diaries. Newborn cord blood DNAm levels of 850K CpG sites were measured using the Illumina Infinium HumanMethylationEPIC array. Associations of each biomarker, dietary index and intervention with DNAm were examined.

**Results:**

Early pregnancy HOMA-IR and HOMA-%B were associated with lower DNAm at CpG sites cg03158092 and cg05985988, respectively. Early pregnancy insulin sensitivity was associated with higher DNAm at cg04976151. Higher late pregnancy insulin concentrations and GL scores were positively associated with DNAm at CpGs cg12082129 and cg11955198 and changes in maternal GI with lower DNAm at CpG cg03403995 (Bonferroni corrected *P* < 5.99 × 10^−8^). These later associations were located at genes previously implicated in growth or regulation of insulin processes. No effects of the intervention on cord blood DNAm were observed. None of our findings were replicated in previous studies.

**Conclusion:**

Among women who were overweight or obese, maternal pregnancy dietary glycemic indices, glucose, and insulin homeostasis were associated with modest changes in their newborn methylome.

**Trial registration:**

ISRCTN29316280

Maternal obesity is associated with pregnancy complications that are likely to affect fetal growth and offspring long-term health (1–3). Numerous studies have reported higher risk of gestational diabetes mellitus (GDM) with increasing weight or body mass index (BMI) (4). Consequently, GDM is associated with adverse pregnancy outcomes, infant macrosomia (5), and higher risk of long-term metabolic disease and development of type 2 diabetes mellitus (DM) in offspring (6). These associations have also been observed with elevated maternal glucose concentrations below the GDM threshold (7). Epigenetic modifications are plausible mechanisms linking the in utero environment and later disease risk (8). DNA methylation (DNAm) is the most-studied epigenetic modification and has a critical role in the control of gene expression throughout development (9). Several studies, using candidate-gene and genome-wide approaches, have reported significant changes in the child's epigenome with exposure to maternal GDM (8). A recent meta-analysis showed that maternal GDM was associated with lower cord blood methylation levels within 2 regions, one of which is upregulated in type 1 and type 2 DM (10). Impaired insulin secretion and increased insulin resistance (IR) [or decreased insulin sensitivity (IS)] play a major role in the pathogenesis of DM (11–13). Inadequate pancreatic β-cell function is also involved in the development of hyperglycemia and DM (11). However, few studies have investigated impaired maternal glucose metabolism with the infant's epigenome in a high-risk population of women with obesity (14, 15).

Improving dietary and lifestyle behaviors could help reduce adverse pregnancy outcomes in women of reproductive age (16). Pregnancy is a unique period of change when women are likely to be engaged and motivated to make healthier choices to benefit the health of their baby (17). One randomized controlled trials (RCTs) conducted so far, UK Pregnancies Better Eating and Activity Trial (UPBEAT), has focused on the impact of an antenatal lifestyle intervention in pregnant women who were overweight or obese and showed that the intervention in pregnancy appeared to attenuate GDM and 1-h and 2-h plasma glucose associated methylation changes in cord blood (14).

The Pregnancy Exercise And nutrition Research Study (PEARS) is a RCT of a diet and exercise lifestyle intervention with a smartphone application support to prevent GDM in pregnant women who are overweight or obese (18). Here, utilizing samples from the PEARS trial, we sought to (1) identify associations between prenatal maternal glycemia and insulinemic status [including early and late pregnancy fasting glucose and insulin concentrations and derived indices of IR (HOMA-IR), IS (QUICKI), and β-cell function (HOMA-%B), 1-h and 2-h glucose concentrations following an oral glucose tolerance test (OGTT), and (GDM)] dietary glycemic index (GI) and glycemic load (GL), and offspring cord blood DNAm and (2) investigate whether a lifestyle intervention for pregnant women who were overweight or obese was associated with methylation changes.

## Materials and Methods

### Study Design

The PEARS study is a single-center RCT carried out at the National Maternity Hospital, Dublin, Ireland (ISRCTN29316280). Details of the full study protocol, including eligibility criteria, recruitment and enrollment, and data collection, have been previously published (19). The study received ethical approval from the National Maternity Hospital Ethics Committee, and written informed maternal consent was obtained. This was a RCT of a mobile health behavioral lifestyle intervention with a smartphone app support to prevent GDM in pregnant women who were overweight or obese. Briefly, between 2013 and 2016, 565 women were recruited at their first antenatal visit. Eligibility criteria included: <18 weeks’ gestation, singleton pregnancy, 18 to 45 years of age, BMI ≥ 25 kg/m^2^ and ≤ 39.9 kg/m^2^, and in possession of a smartphone. Exclusion criteria were: previous GDM and any medical condition requiring treatment, multiple pregnancy, or not in possession of a smartphone. Participants returned for their first study visit within 2 weeks for randomization to either the intervention or control group. A biostatistician created a computer-generated random sequence, and participants were then randomized in a 1:1 ratio to the intervention or control group. Allocation was concealed in sequentially numbered, sealed, opaque envelopes. Randomized participants were stratified by BMI to ensure equal numbers of overweight and obese women in each group. Neither participants nor researchers were blinded to the intervention or outcomes (18, 19). For the current study, we obtained cord blood samples for women who had full clinical data; as many delivered at night and over weekends when we did not have staff available to collect samples, due to budgetary constraints, this resulted in collection of cord bloods from 186 newborns, from which DNA was extracted and quantified. Following the DNAm quality control and normalization steps (see *DNA Methylation Assessment*), 172 subjects were included in the analyses. This study is reported as per the CONSORT 2010 guideline [see Supplementary Appendix A (20)].

### Intervention

Participants allocated to the intervention group received standard antenatal care plus a “Healthy Lifestyle Package” with a single face-to-face education session, delivered by a research dietitian or nutritionist. The dietary advice focused on achieving a low GI diet and included additional advice on portion sizes of carbohydrates and general healthy eating for pregnancy recommendations. The education was equicaloric so did not promote weight loss (21). An exercise prescription of 30 minutes of moderate exercise 5 to 7 days per week was given by the obstetrician. Participants were advised to have an appropriate gestational weight gain in accordance with the Institute of Medicine guidelines (22). Women in the intervention arm were also provided with access to the study-specific smartphone app, received emails every 2 weeks (sent by the research team), and had 2 follow-up face-to-face hospital visits at 28 and 34 weeks’ gestation (22), all underpinned by behavior change theory. Women randomized to the control group received standard antenatal care, which does not include consistent dietary, physical activity, or gestational weight gain advice (19). Women in the intervention group had significantly reduced GL scores and increased physical activity levels compared with women in the control group. No significant differences were observed for GDM or glucose and insulin levels in the main population (18).

### Data Collection and Classification of Variables

#### Maternal glycemia and insulinemic status

Maternal blood samples were collected at the baseline visit and the study follow-up (28 weeks pregnancy) after at least 8 hours of an overnight fast. At the shortest possible interval post-venepuncture, blood samples were centrifuged at 3000 rpm for 10 minutes, and aliquots were stored at −80°C pending analysis. Plasma glucose was analyzed using the AU680 Chemistry analyser (Beckman Coulter Inc., High Wycomb, UK) and the hexokinase method. Insulin was quantified by automated immunoassay (Roche, catalog no. 120175471, RRID: AB_2756877*Updated) with typical CVs <5% (22). Participants had also a 2-hour OGTT performed according to the International Association of Diabetes and Pregnancy Study Groups criteria (23) with glucose and insulin analyzed as described previously. GDM was confirmed if at least 1 glucose value was at or above the following: fasting ≥5.1, 1-h ≥10, and 2-h ≥8.5 mmol/L (23). The homeostatic model assessment (HOMA) was used to assess estimates of IR and β-cell function from basal fasting plasma glucose (FPG) and insulin (FPI) concentrations (24) using the following equations: HOMA1-IR = (FPI (mU/L) * FPG (mmol/L))/22.5 and HOMA1-%B = (20 * FPI)/(FPG − 3.5). Higher HOMA-IR values reflect greater insulin resistance. Quantitative insulin sensitivity check index (QUICKI) was used to determine IS as follows: QUICKI = 1/(log_10_(FPI) (mU/L) + log_10_(FPG) (mg/dL)) (25). Lower QUICKI values reflect low IS. A previous publication showed that these fasting-derived measures correlate reasonably well with a direct measurement of IS using the hyperinsulinemic-euglycemic clamp (r = 0.6 to 0.7) (26). These findings have been confirmed in pregnant women (27). Another study showed that obese subjects exhibited strong correlations with fasting measures of IS (r = 0.65) (28). However, it has been reported that HOMA-B is poorly correlated with intravenous glucose tolerance test measures of insulin secretion (r = 0.33)—especially in pregnancy (29). Absolute changes between early and late pregnancy concentrations of fasting glucose (Δglucose), insulin (Δinsulin), HOMA (ΔHOMA), and QUICKI (ΔQUICKI) values were calculated and used in further analyses, with positive values indicating an increase and negative values a decrease of the previous markers.

#### Calculation of dietary glycemic indices

Maternal diet was assessed at baseline and 28 weeks’ gestation visits using 3-day food diaries including 1 weekend day. The types and amount of food consumed were recorded by participants in household measures (eg, teaspoons, tablespoons) or using actual weight listed. Data were entered into Nutritics Professional Nutrition Analysis Software, version 4.267, Research Edition (Nutritics, Dublin, Ireland, www.nutritics.com). GL and GI were calculated. The GI describes the blood glucose response after consumption of a carbohydrate-containing test food relative to a carbohydrate-containing reference food and can differentiate the potential of carbohydrate-rich food to affect blood glucose (30). The GL, the product of GI and total dietary carbohydrate intake, takes into account the amount eaten and provides a useful measure to assess the overall glycemic effect of the diet (30). Variations between early and late GI (ΔGI) and GL scores (ΔGL) were calculated.

#### Maternal and child information

On providing consent, mothers’ height and weight were measured by a relevant healthcare professional at early pregnancy. Weight was measured to the nearest 0.1 kg in light clothing using a SECA weighing scale (SECA GmbH & co. kg., Hamburg, Germany). Height was measured to the nearest 0.1 cm using a wall-mounted stadiometer after removal of footwear (31). Height at baseline and early pregnancy weight were used to calculate maternal BMI (kg/m²). Maternal age, ethnicity (White/others), and education level (no schooling/primary/secondary vs partial/complete third-level education) were collected at baseline. Smoking status during pregnancy (yes/no) was combined using smoking status at early and late pregnancy. Additional information related to pregnancy including parity (coded as primiparous if no previous deliveries, multiparous if ≥ 1 previous delivery), child sex, gestational age at birth, and birthweight were collected from medical charts at delivery.

### DNA Methylation Assessment

Cord blood samples were collected at delivery into EDTA blood collection tubes and stored at −80°C. Description of the DNA extraction is provided in Supplementary Appendix B (20). The DNA quality control and DNAm analysis steps were performed by the Bristol Bioresource Laboratories (Bristol, UK). The Illumina Infinium® HumanMethylation EPIC BeadChip assay (EPIC array) was used to evaluate the methylation status of 865 859 individual CpG sites in the cord blood samples (n = 186) (Illumina Inc., CA). Arrays were scanned using an Illumina iScan. Hybridization/fluorescence signals were written to idat files, and initial quality review was assessed using GenomeStudio. We performed quality control and normalization of the methylation data using the *meffil* R package as described by Min et al (32). In the quality-control step, we excluded samples with multiple control probe outliers and samples with sex mismatches (n = 14 of 186 samples). We excluded probes targeting the sex chromosomes, probes with a bead count <3 in >10% of samples, and probes having a detection *P*-value >0.01 in >10% of samples. The remaining 834 792 CpG site probes were included in downstream analyses. Samples were normalized by using functional normalization as implemented in the *meffil* R package. Probe intensity quantiles were adjusted with the 15 first control probe principal components. Normalized methylation levels or β-values at CpG sites were estimated as ratios of the methylated probe intensity to the overall probe intensity (sum of methylated and unmethylated probe signals) ranging from 0 to 1.

### Statistical Analysis

Characteristics of the population selected are described with mean ± SD and numbers (%). Continuous variables were assessed for normality through visual inspection of histograms, the Shapiro-Wilk test for normality, and inspection of variance homogeneity. Student *t*-tests (independent samples) and the Wilcoxon rank test or (Mann-Whitney *U* test) for nonnormally distributed variables were used to compare characteristics between control and intervention. For categorical variables, the Chi-squared test was used. Correlations between glucose and insulin markers were assessed using Pearson's correlation coefficient and tested for difference from the null using a *t*-test.

To avoid undue influence of extreme outliers, maternal insulinemic and glycemic biomarker values >4 SD from the population mean were excluded (exclusion of 1 to 3 samples), based on the statistical convention that these observations can be considered to be “far outliers” (33).

#### Epigenome-wide association study

Using the *meffil package*, we conducted an epigenome-wide association study (EWAS) to identify associations between maternal glycaemia and insulinemic status (including early and late pregnancy and variations of fasting glucose and insulin concentrations and derived indices of IR (HOMA-IR), IS (QUICKI), and β-cell function, 1-h and 2-h glucose concentrations following an OGTT, and GDM], dietary glycemic indices (GI and GL), intervention, and DNAm in cord blood. We used variables with international (SI) units in pmol/L for insulin and mmol/L for glucose levels. Associations were adjusted for potential confounders identified from the literature and technical factors related to methylation measurement. Two models were considered: a first “basic model” adjusted for child sex, maternal smoking during pregnancy, gestational age at birth (continuous), batch effect (represented by the sample plate variable), and cell-type proportions. Cell type proportions were estimated from DNAm using reference methylomes derived from cord blood. Cell types included Bcell, granulocytes, natural killer, monocytes, CD4+ T-lymphocytes, CD8+ T-lymphocytes, and nucleated red blood cells (34). A second “full model” adjusted additionally for parity, maternal education (third level or not), ethnicity (White/others), maternal age, maternal BMI, and birthweight. Lastly, we evaluated whether the magnitude of the effect estimates for the loci were similar in the control group and whether the antenatal lifestyle intervention might modify the methylation levels.

The percentage of missing data associated with maternal smoking was around 7%, so we estimated smoking effects using the methylation levels of the AHRR CpG site cg05575921, a site strongly associated with maternal smoking during pregnancy across multiple studies (35) including in our own data (*P-*value = 1.8 × 10^−10^). Statistical significance was assessed using a *P*-value threshold corrected for multiple tests (*P* < 5.99 × 10^−8^ = 0.05/834 792) (36). DNAm outliers were omitted from analyses using an automatic trimming method that excluded data points below the 25th percentile minus 3 interquartile ranges or above the 75th percentile plus 3 interquartile ranges for each probe. After identification of the CpG site associations, the integrative gene database Genecards (37) was used to describe functions of the identified genes. We also explored the genetic basis of DNAm variation by looking up single nucleotide polymorphisms (SNPs) associated with CpG sites and methylation QTL (mQTL) identified in the largest analysis to date (38).

#### Look-up of previously identified CpGs

A look-up analysis of CpGs previously associated with maternal GDM, hyperglycemia, or hyperinsulinemia was conducted. Look-ups included associations with maternal dysglycemia reported in the UPBEAT RCT, a lifestyle intervention (low GI diet plus physical activity) in pregnant women with obesity (14), a meta-analysis of maternal GDM and cord blood DNAm by the Pregnancy and Childhood Epigenetics consortium (PACE), which included 7 pregnancy cohorts (10), associations of maternal pregnancy glucose and insulin concentrations with newborn DNA methylation in the Generation R study (15), and, finally, a recent meta-analysis that included 8 birth cohorts (39). Statistical significances of look-ups were corrected for multiple tests using Bonferroni-corrected *P*-value thresholds.

#### Replication

Replication of maternal GI and GL results was accomplished by analyzing DNAm measurements using the EPIC array in child saliva at age 9 in 244 mother-child pairs in the Lifeways Cross-Generation Cohort Study. Lifeways is a prospective family study whose objective is to document diet and lifestyle (40) that recruited 1124 mothers between 2001 and 2003 in 2 maternity hospitals in the Republic of Ireland. Written informed consent was obtained for all participants, and ethical approval was granted by ethics committees of the Coombe Womens and Infants University Hospital, Dublin, University College Dublin, and the Irish College of General Practitioners and University College Hospital, Galway, Ireland. Associations were adjusted for the same covariates as listed previously for discovery analyses. Habitual dietary intakes of the women during the first trimester of pregnancy were assessed at inclusion (12 to 16 weeks of gestation) using a validated 149-item semiquantitative food frequency questionnaire (41). Insulinemic index (II) and insulinemic load (IL) were also available (42). The II and its corresponding insulinemic load IL measure “insulin demand” elicited by various foods [details in Supplementary Appendix B (20)]. EPIC array data were normalized using the functional normalization method implemented in the R package *meffil* (32) as described previously [Supplementary Appendix B (20)]. We excluded samples showing evidence of extreme values for the dietary scores defined as values >4 SD from the mean (1 sample for GL and 2 samples for IL). DNAm outliers were identified using the same automatic method described earlier.

## Results

### Population Characteristics

The characteristics of the families selected for analysis are summarized in Table 1. Mean maternal age was 33 (±4.5) years. Most mothers (87.4%) had a tertiary education level. About 64% of mothers were overweight and 36% were obese at recruitment, and 7.5% smoked during pregnancy. Gestational diabetes was diagnosed for 18 women.

**Table 1.** Comparison of maternal characteristics between the intervention and control group

	N	Population % (n)	Missing data % (n)	Control group, n = 76	Intervention group, n = 96	*P*-value
Maternal characteristics						
Maternal age	171	32.72 ± 4.5	0.58 (1)	32.18 ± 4.06	33.15 ± 4.83	0.16
Maternal ethnicity (White)	170	90.6 (154)	1.16 (2)	88 (66)	92.63 (88)	0.30
Maternal education	167		2.91 (5)			0.58
Below tertiary		12.6 (21)		10.96 (8)	13.83 (13)	
Tertiary or above		87.4 (146)		89.04 (65)	86.17 (81)	
Maternal BMI (kg/m²)^*a*^	172	29.78 ± 3.5	0 (0)	29.89 ± 3.55	29.7 ± 3.46	0.76
Baseline BMI category	172		0 (0)			0.61
Overweight		64 (110)		61.84 (47)	65.63 (63)	
Obesity		36 (62)		38.16 (29)	34.38 (33)	
Smoking during pregnancy (yes)	160	7.5 (12)	6.98 (12)	8.7 (6)	6.59 (6)	0.62
Physical activity at baseline^*a*^ (MET-minutes per week)	148	465.23 ± 439.2	13.95 (24)	508.94 ± 459.32	431.93 ± 422.94	0.26
Physical activity at 28 weeks^*a*^ (MET-minutes per week)	125	497.89 ± 383.2	27.33 (47)	418.13 ± 345.15	560.56 ± 402	0.09
Glucose and insulin measures						
Fasting glucose at baseline, mmol/L^*a*^	154	4.54 ± 0.3	10.46 (18)	4.52 ± 0.26	4.55 ± 0.33	0.46
OGTT results at 28 weeks of gestation (mmol/L)^*b*^						
Fasting glucose	169	4.44 ± 0.3	1.74 (3)	4.46 ± 0.35	4.43 ± 0.35	0.61
Glucose 1-h pp	170	7.21 ± 1.8	1.16 (2)	7.05 ± 1.78	7.34 ± 1.83	0.30
Glucose 2-h pp^a^	169	5.65 ± 1.3	1.74 (3)	5.56 ± 1.3	5.72 ± 1.37	0.48
Δ Fasting glucose^*a*^	152	−0.11 ± 0.3	11.63 (20)	−0.07 ± 0.26	−0.14 ± 0.33	0.06
Maternal GDM (Yes)	170		1.16 (2)			0.64
Yes		10.6 (18)		9.33 (7)	11.58 (11)	
No		89.4 (152)		90.67 (68)	88.42 (84)	
Insulin at baseline,^*a*^ pmol/L	161	57.68 ± 24.8	6.39 (11)	56.26 ± 25.1	58.86 ± 24.59	0.33
Insulin at 28 weeks,^*a*^ pmol/L	159	81.95 ± 40.9	7.56 (13)	86.65 ± 46.4	77.85 ± 35.23	0.45
Δ Insulin^*a*^	150	22.15 ± 27.2	12.79 (22)	27.36 ± 31.2	17.46 ± 22.26	0.14
HOMA1-IR index at baseline^*a*^	144	1.94 ± 0.9	16.28 (28)	1.91 ± 0.95	1.96 ± 0.9	0.58
HOMA1-IR index at 28 weeks^*a*^	158	2.68 ± 1.3	8.14 (14)	2.88 ± 1.57	2.5 ± 1.05	0.29
Δ HOMA1-IR^*a*^	134	0.67 ± 0.9	22.09 (38)	0.86 ± 1.02	0.5 ± 0.85	0.10
HOMA1-%B at baseline^*a*^	143	190.29 ± 93.2	16.86 (29)	195.25 ± 106.14	186.05 ± 81.03	0.84
HOMA1-%B at 28 weeks^*a*^	157	312.75 ± 183.6	8.72 (15)	324.58 ± 195.99	302.47 ± 172.69	0.57
Δ HOMA1-%B^*a*^	134	119.71 ± 158.5	22.09 (38)	139.81 ± 150.37	101.33 ± 164.54	0.33
QUICKI index at baseline^*a*^	144	0.35 ± 0.03	16.28 (28)	0.35 ± 0.03	0.35 ± 0.03	0.41
QUICKI index at 28 weeks^*a*^	159	0.34 ± 0.02	7.56 (13)	0.33 ± 0.02	0.34 ± 0.02	0.50
Δ QUICKI index	134	−0.016 ± 0.02	22.09 (38)	−0.02 ± 0.02	−0.01 ± 0.02	0.14
Dietary glycemic indices						
Energy intake (kcals/day) at baseline^*c*^	139	1868.65 ± 408.2	19.19 (33)	1902.67 ± 395.35	1844.28 ± 417.85	0.41
Energy intake (kcals/day) at 28 wk^*c*^	119	1796.99 ± 405.8	30.81 (53)	1892.87 ± 415.97	1720 ± 383.45	0.02
Glycemic index at baseline	139	58.97 ± 4.7	19.19 (33)	58.34 ± 4.34	59.42 ± 4.97	0.19
Glycemic index at 28 weeks	119	57.93 ± 4.2	30.81 (53)	58.66 ± 3.58	57.34 ± 4.56	0.09
Δ Glycemic index	108	−1.23 ± 5.7	37.21 (64)	0.76 ± 5.5	−2.82 ± 5.36	<0.01
Glycemic load at baseline^*a*^	139	135.59 ± 32.8	19.19 (33)	139.62 ± 31.03	132.7 ± 33.84	0.31
Glycemic load at 28 weeks^*a*^	119	127.36 ± 31.9	30.81 (53)	135.99 ± 33.91	120.44 ± 28.7	<0.01
Δ Glycemic load	108	−9.3 ± 33.6	37.21 (64)	−3.78 ± 35.28	−13.72 ± 31.73	0.13
Pregnancy information						
Gestational age of recruitment (wk)^*a*^	171	14.89 ± 1.7	0.58 (1)	14.97 ± 1.89	14.82 ± 1.61	0.43
Gestational age at birth (wk)^*a*^	172	40.13 ± 1.4	0 (0)	40.15 ± 1.41	40.11 ± 1.39	0.73
Primiparous (yes)	172	47.1 (81)	0 (0)	51.32 (39)	43.75 (42)	0.32
Birth weight (g)	172	3614.1 ± 517.6	0 (0)	3658.95 ± 490.97	3578.59 ± 537.61	0.31

Data presented as mean ± SD for continuous variables or % (n) for categorical variables. Description of the glucose/insulin markers are presented after exclusion of extreme values >4 SD from the average mean. *P*-values are derived from Chi-squared test for categorical variables and sample *t*-tests for continuous variables unless otherwise noted. Significant *P-*value <0.05. Baseline refers to data collected between 14 and 16 weeks weeks’ gestation.

Abbreviations: GDM, gestational diabetes mellitus; HOMA1-IR, homeostatic model assessment; HOMA1-%B, B cell function; OGTT, oral glucose tolerance test; pp, postprandial; QUICKI, quantitative insulin sensitivity check index.

a *P*-value used the Mann-Whitney *U* test.

b Glucose tolerance test based on Hyperglycemia and Adverse Pregnancy Outcome criteria.

c Energy intakes are between 900 and 3000 kcals/days and 830 and 3000 kcal/d at early and late pregnancy, respectively.

The comparison of characteristics between the intervention (n = 96) and control (n = 76) groups did not reveal any significant differences in maternal sociodemographic characteristics (Table 1). At baseline, there were no differences between intervention and control groups for mean daily GI and GL intakes. However, GL, GI scores, and energy intakes were lower in the intervention group in late pregnancy (postintervention) (*P*-values <0.01 and = 0.02, respectively). Glucose and insulin concentrations and physical activity levels were similar between the 2 groups at early and late pregnancy in the subgroup examined for this analysis. The comparison of characteristics between families who had epigenetic data and those who did not show no major differences, except a higher maternal BMI in our selected population [Supplementary Table S1 (20)].

Correlations between the glucose biomarkers and dietary glycemic indices, and between the insulin biomarkers and indices of insulin resistance, insulin sensitivity, and β-cell function, were evaluated [Supplementary Figs. S1 and S2 (20)]. Glucose 1-h postprandial (pp) and glucose 2-h pp were strongly correlated: r = 0.67 (*P* < 0.001). However, correlations between dietary glycemic indices and glucose biomarkers were weak (|r| < 0.20). As expected, the correlation between insulin and HOMA1-IR index was strong (r = 0.99 at baseline, *P* < 0.001), while there was a negative correlation between insulin resistance and insulin sensitivity (r = −0.90, *P* < 0.001). Correlation between indices of insulin resistance and β-cell function was slightly lower (r = 0.64, *P* < 0.001).

### Epigenome-wide Association Studies

#### Maternal glycaemia, insulinemic status, and indices of insulin resistance, insulin sensitivity, and β-cell function

**Figure 1.**
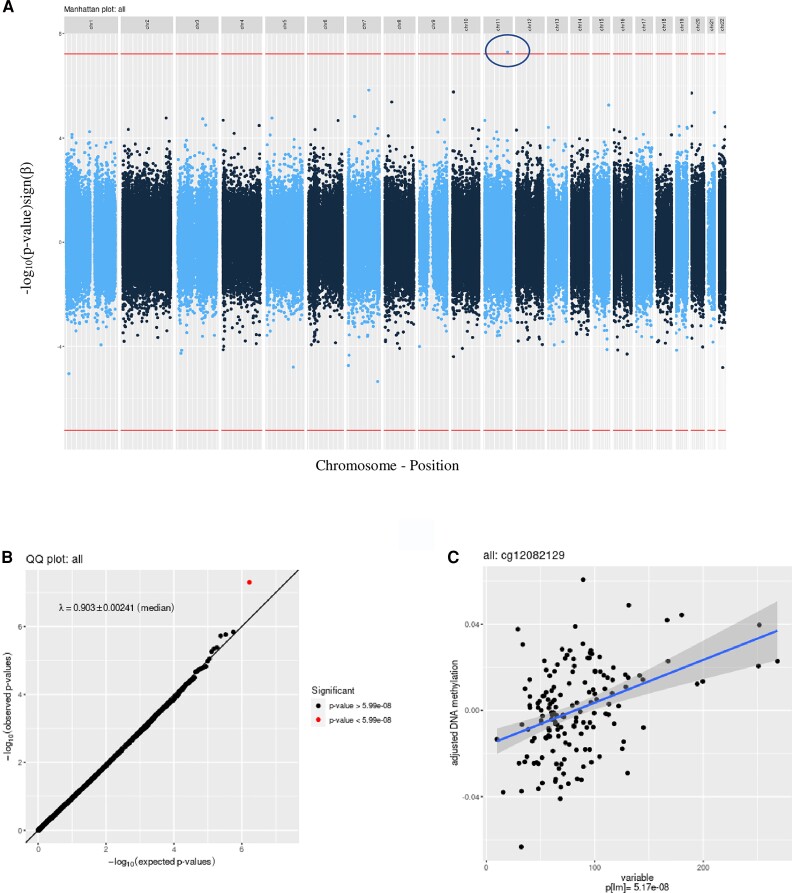
Manhattan plot, quantile-quantile plot, and CpG plot of epigenome-wide association results associated with the maternal insulin concentrations at late pregnancy in the basic model.

Maternal insulin levels at late pregnancy were initially associated with 3 CpG sites cg07719492 (*P-*value = 2.16 × 10^−8^), cg19816930 (*P-*value = 3.16 × 10^−8^), and cg12082129 (*P*-value = 5.12×10^−8^); however, the first 2 CpG sites associations were driven by methylation outliers. After removing outliers (see *Methods*), only the association with cg12082129 persisted (β = 0.0002 per 1 unit increase in the score, *P-*value = 5.04 × 10^−8^ in the basic model) (Table 2). This CpG is located in the first intron of the proprotein convertase subtilisin/kexin type 7 (*PCSK7*) gene and <1000 bp upstream of the ring finger protein 2014 (*RNF2014*) gene. It is associated with 27 SNPs; the top 1 rs2252145 is located <200 bp away and is associated with 13% reduction in DNAm. No associations between maternal insulin levels at early pregnancy or insulin variations throughout pregnancy survived correction for genome-wide tests. Manhattan plots, quantile–quantile plot, and CpG plot of genome-wide association statistics are shown in Figure 1.

(A) Manhattan plot: the red dashed line represents Bonferroni correction significance threshold (*P* = 5.99 × 10^−8^). The direction of the associations is reported with negative effects below zero and positive effects above zero. Model adjusted for child sex, gestational age, maternal smoking during pregnancy, batch effect, and estimated cell-type proportions. (B) Quantile-quantile plot of epigenome-wide association results associated with maternal late insulin in the basic model. (C) CpG plot of the CpG site associated with maternal late insulin in the basic model.

**Table 2.** Associations between maternal estimates of insulin resistance (HOMA-IR), insulin sensitivity (QUICKI), β-cell function (HOMA-%B), and dietary glycemic indices with cord blood DNA methylation

	Basic model^*a*^									Full model^*d*^	
	Probe ID	Probe mean (SD)	β (95% CI)^*b*^	*P*-value	Chr	Position	Gene	Gene region	SNPs^*c*^	Coefficient β	*P-*value
Maternal insulin concentrations at 28 weeks^*e*^	cg12082129	0.14 (0.02)	0.0002 (0.0002; 0.0003)	5.04E−08	chr11	117102463	RNF214; PCSK7; RNF214	TSS1500; 5′UTR; TSS1500	FALSE	0.0002	4.25E−06
Maternal early HOMA1-IR score^*e*^	cg03158092	0.77 (0.07)	−0.0179 (−0.0248; −0.011)	1.37E−06	chr22	32968912	SYN3; SYN3; SYN3	Body; Body; Body	FALSE	−0.0229	5.99E−08
Maternal early HOMA1-%B score^*e*^	cg05985988	0.58 (0.08)	−0.0003 (−0.0005; −0.0002)	5.45E−08	chr6	15304885	JARID2	Body	TRUE	−0.0003	2.19E−06
Maternal early QUICKI score^*f*^	cg04976151	0.29 (0.05)	0.7673 (0.3870; 1.1475)	1.09E−04	chr16	57496328	POLR2C	TSS1500	FALSE	1.1467	5.11E−08
Maternal GL at 28 weeks	cg11955198	0.32 (0.05)	0.0007 (0.0005; 0.001)	2.59E−08	chr11	1918783	NA	NA	FALSE	0.0008	3.39E−08
Maternal Δ GI	cg03403995	0.65 (0.07)	−0.0054 (−0.0073; −0.0035)	1.74E−07	chr15	28020621	OCA2; OCA2	Body; Body	FALSE	−0.0058	3.99E−08

Statistical significance was assessed using a p-value threshold corrected for multiple tests (*p* < 5.99 × 10^−8^). Abbreviations: HOMA1-IR, homeostatic model assessment; HOMA1-%B, B cell function; QUICKI, quantitative insulin sensitivity check index.

a Basic model: adjusted for child sex, gestational age, maternal smoking during pregnancy, batch effect, estimated cell-type proportions. Maternal insulin at 28 weeks (n = 159), maternal early HOMA1-IR (n = 144), maternal early HOMA1-%B (n = 143), maternal early QUICKI (n = 143), maternal GL at 28 weeks (n = 119), maternal Δ GI (n = 108).

b β values are increase of methylation expressed per 1-point increase in the glycemic, IR, IS, QUICKI scores, per 1 unit in the insulin concentration (pmol/L).

c SNP equal to TRUE for any probes that contain a common SNP with minor allele frequency >0.01.

d Full model: model adjusted for child sex, gestational age, maternal smoking during pregnancy, batch effect, estimated cell-type proportions, parity, maternal education, maternal age, body mass index, ethnicity, and birthweight. Maternal insulin at 28 weeks (n = 152), maternal early HOMA-IR (n = 138), maternal early HOMA1-%B (n = 137), maternal early QUICKI (n = 137), maternal GL at 28 weeks (n = 118), maternal Δ GI (n = 107).

e Models with exclusion of methylation outliers using the interquartile range method.

f Model with exclusion of 1 sample showing methylation outliers.

Maternal early HOMA-IR was associated negatively with 1 CpG site cg03158092 (β = −0.0229 per 1-point increase in the score, *P-*value = 5.99 × 10^−8^), located near the synapsin III (*SYN3*) gene in the fully adjusted model [Table 2 and Supplementary Fig. S3 (20)]. Secondly, we found that early maternal HOMA1-B% was initially associated with 8 CpG sites, although all but 1 association was driven by methylation outliers. This association was with the cg05985988 CpG site (β = −0.0003 per 1-point increase in the score, *P-*value = 5.45 × 10^−8^), located near the jumonji and at-rich interaction domain containing 2 (*JARID2*) gene [Table 2 and Supplementary Fig. S4 (20)]. We observed 1 association with early maternal QUICKI score but only in the fully adjusted model with cg04976151 (β = 1.15 per 1-point increase in the score, *P-*value = 5.11 × 10^−8^), on the RNA polymerase II subunit C (*POLR2C*) gene [Table 2 and Supplementary Fig. S5 (20)]. Several SNPs are associated with cg04976151 (n = 261); the strongest, rs11076191, located about 60Kb away, is associated with a 30% increase of methylation.

Finally, no associations between maternal GDM, the FPG at early and late pregnancy, or 1-h and 2-h glucose concentrations following an OGTT survived adjustment for multiple tests.

#### Maternal GI and GL

Maternal GL scores in late pregnancy were associated positively with DNAm at 1 CpG site (cg11955198) (β = 0.0007, per 1-point increase in the score, *P*-value = 2.6 × 10^−8^) in the basic model [Table 2 and Supplementary Fig. S6 (20)]. The association persisted for the full model as well (*P-*value = 3.4 × 10^−8^). Several SNPs (n = 229) are known to be associated with cg11955198, and the top one, rs11600982, located about 60 Kb away, is associated with a 30% reduction of methylation of this CpG.

Maternal GI changes during pregnancy was associated with DNAm at 1 CpG site (cg03403995) located near the OCA2 melanosomal transmembrane protein (*OCA2*) gene (β = −0.0058 per 1-point increase in the score, base model *P*-value = 1.74 × 10^−7^, full model *P*-value = 4.0 × 10^−8^) [Supplementary Fig. S7 (20) and Table 2].

#### Intervention

We found that the intervention itself was not associated with strong effects in cord blood DNAm. Table 3 shows the 10 most strongly associated CpG sites; none survive adjustment for genome-wide tests. Furthermore, we found that the GL, GI, and insulin associations with DNAm evaluated in the overall population were identical to those in the control group (Table 4).

**Table 3.** Associations between the intervention and the top 10 individual CpG sites

Basic model^*a*^								Full model^*c*^	
ProbeID	Probe mean (SD)	β (95% CI)^*b*^	*P*-value	Chromosome	Position	Gene symbol	Common SNPs	Coefficient	*P*-value
cg07456470	0.80 (0.04)	−0.0305 (−0.0427; −0.0183)	2.46E−06	chr21	43977377	SLC37A1	True	−0.0327	1.70E−06
cg03472039	0.58 (0.06)	0.0372 (0.0223; 0.0521)	2.46E−06	chr2	86842330	RNF103; RNF103; RNF103-CHMP3	False	0.0380	4.89E−06
cg04096472	0.07 (0.02)	0.0105 (0.0061; 0.0149)	5.79E−06	chr7	137687229	CREB3L2	False	0.0094	4.24E−05
cg08502797	0.12 (0.03)	−0.0148 (−0.0212; −0.0085)	8.72E−06	chr1	216896593	ESRRG; ESRRG	False	−0.0160	4.30E−06
cg01875764	0.81 (0.04)	0.0241 (0.0137; 0.0346)	1.12E−05	chr6	31760288	VARS	False	0.0247	3.53E−05
cg20467412	0.52 (0.08)	−0.0495 (−0.0711; −0.0279)	1.31E−05	chr19	5799340	NA	True	−0.0498	3.69E−05
cg24305685	0.07 (0.02)	−0.0119 (−0.0171; −0.0066)	1.68E−05	chr12	116715986	MED13L	False	−0.0114	4.42E−05
cg20250426	0.15 (0.03)	−0.0184 (−0.0266; −0.0102)	2.06E−05	chr7	96655105	DLX5	False	−0.0192	3.47E−05
cg21935511	0.31 (0.03)	−0.0171 (−0.0248; −0.0094)	2.26E−05	chr17	74523449	PRCD; CYGB	False	−0.0177	3.02E−05
cg25530613	0.41 (0.06)	0.0332 (0.0183; 0.0481)	2.27E−05	chr5	63868125	RGS7BP	False	0.0331	6.37E−05

SNP equal to TRUE for any probes that contain a common SNP with minor allele frequency >0.01.

a Basic adjusted model: adjusted for child sex, gestational age, maternal smoking during pregnancy, batch effect, estimated cell-type proportions (n = 172).

b β 95% CI difference in newborn DNA methylation and 95% CI in comparison of the intervention with the control group.

c Fully adjusted model: model adjusted for child sex, gestational age, maternal smoking during pregnancy, batch effect, estimated cell-type proportions, parity, maternal education, maternal age, body mass index, ethnicity, and birthweight (n = 164).

**Table 4.** EWAS epigenome-wide association study associations in the whole-study population and the control arm

	All population									Control arm^*c*^		
	Probe ID	Probe mean (SD)	Effect	*P*-value	Chr	Position	Gene	Gene region	SNPs	Probe mean (SD)	Effect	*P-*value
Maternal Insulin at 28 weeks^*a*^	cg12082129	0.14 (0.02)	0.0002	5.04E−08	chr11	117102463	RNF214; PCSK7; RNF214	TSS1500; 5′UTR; TSS1500	False	0.15 (0.02)	0.0003	1.35E−05
Maternal GL at 28 weeks^*a*^	cg11955198	0.32 (0.05)	0.0007	2.59E−08	chr11	1918783	NA	NA	False	0.33 (0.05)	0.0007	7.2E−04
Maternal Δ GI^*b*^	cg03403995	0.65 (0.07)	−0.0058	3.99E−08	chr15	28020621	OCA2; OCA2	Body; Body	False	0.65 (0.07)	−0.007	4.6E−04

Effects per 1-point increase in the glycemic scores, per 1 unit in the insulin concentration (pmol/L). Statistical significance was assessed using a p-value threshold corrected for multiple tests (*p* < 5.99 × 10^−8^).

Abbreviations: EWAS, epigenome-wide association study; GI, glycemic index; GL, glycemic load; NA, not available.

a Basic adjusted model: adjusted for child sex, gestational age, maternal smoking during pregnancy, batch effect, and estimated cell-type proportions.

b Fully adjusted model: model adjusted for child sex, gestational age, maternal smoking during pregnancy, batch effect, estimated cell-type proportions, parity, maternal education, maternal age, body mass index, ethnicity, and birthweight.

c Control arm: maternal GL at 28 weeks; model 1 (n = 53), maternal Δ GI; model 2 (n = 47), maternal insulin concentrations at 28 weeks; model 1 (n = 74).

#### Look-up analyses

None of the previously reported associations survived adjustment for multiple tests in our study. Supplementary Tables S2, S3, S4, and S5 (20) show summary statistics for associations reported in the UPBEAT RCT EWAS of fasting glucose, glucose 1-h pp, glucose 2-h pp, and maternal GDM (with additional adjustment for baseline BMI), respectively (14). Supplementary Table S6 (20) shows summary statistics for cord blood CpG methylation associations with maternal GDM reported by the PACE consortium (10). Finally, summary statistics for CpG site associations with early glucose and insulin concentrations in the Generation R study (15) are shown in Supplementary Tables S7 and S8 (20). Supplementary Table S9 (20) shows results of maternal fasting glucose and insulin associations with cord blood DNAm in a PACE meta-analysis (39).

#### Replication analysis in Lifeways cohort

No associations between maternal GI, GL, II, and IL scores measured at early pregnancy and child DNAm at 9 years were reported. Supplementary Table S10 (20) shows the 10 most strongly associated CpG sites with each maternal dietary score in Lifeways from 2 different adjusted models. We also evaluated replication of top associations reported in PEARS within Lifeways but reported no associations after adjustment for multiple tests. Table 5 shows summary statistics of the strongest associations in PEARS between maternal GI and GL and cord blood DNAm alongside corresponding summary statistics in the Lifeways study.

**Table 5.** Comparison of the maternal early GI and GL effects on offspring DNA methylation reported in the PEARS RCT with the Lifeways study

Probe	PEARS EWAS results (n = 136)^*a*^							Lifeways EWAS results (n = 189)^*b*^		
	Probe mean (SD)	Effect	SE	*P*-value	Chr	Position	Gene name	Probe mean (SD)	Effect	*P*-value
Comparison of the maternal GI effects on DNA methylation										
cg16767170	0.81 (0.03)	−0.0028	0.0005	3.14E−07	chr8	87351999	NA	0.80 (0.04)	0.0001	0.85
cg27547993	0.83 (0.07)	−0.0076	0.0015	1.53E−06	chr18	68034154	NA	0.83 (0.10)	0.0003	0.73
cg17190403	0.16 (0.03)	0.0029	0.0006	2.23E−06	chr6	151774260	C6orf211; RMND1	0.19 (0.04)	0.0005	0.14
cg10875957	0.61 (0.08)	0.0067	0.0014	3.87E−06	chr6	41122461	TREML1; TREML1; TREML1	0.53 (0.13)	−0.0021	0.32
cg00610748	0.74 (0.05)	−0.0035	0.0007	4.06E−06	chr13	111935077	ARHGEF7; ARHGEF7; ARHGEF7; ARHGEF7; ARHGEF7	0.55 (0.12)	0.0019	0.27
cg04304696	0.70 (0.09)	−0.0075	0.0016	5.20E−06	chr9	138847903	UBAC1	0.77 (0.13)	0.0021	0.14
cg23655934	0.12 (0.03)	−0.0027	0.0006	6.87E−06	chr3	32823185	NA	0.14 (0.05)	−0.0003	0.67
cg22063391	0.34 (0.07)	0.0063	0.0014	9.14E−06	chr13	53956443	NA	0.31 (0.06)	0.0007	0.54
cg04785083	0.07 (0.08)	−0.0063	0.0014	1.22E−05	chr1	9031262	CA6	0.33 (0.16)	0.0002	0.89
cg14922738	0.40 (0.06)	0.0048	0.0011	1.29E−05	chr22	45335216	PHF21B; PHF21B; PHF21B; PHF21B	0.38 (0.08)	0.0009	0.52
cg13545538	0.54 (0.02)	0.0016	0.0004	1.38E−05	chr7	5567503	ACTB	0.60 (0.03)	0.0003	0.47
cg04670255	0.77 (0.11)	0.0081	0.0018	1.47E−05	chr22	39884829	MGAT3; MGAT3	0.86 (0.10)	−0.0015	0.30
cg22376822	0.92 (0.05)	0.0037	0.0008	1.76E−05	chr11	114112973	ZBTB16; ZBTB16	0.95 (0.02)	0.0001	0.67
cg08902977	0.01 (0.007)	0.0007	0.0001	1.90E−05	chr16	57318244	PLLP	0.02 (0.01)	1.00E−04	0.30
cg18190411	0.35 (0.05)	0.0037	0.0008	1.93E−05	chr10	38989395	ACTR3BP5	0.36 (0.06)	0.0003	0.79
Comparison of the maternal GL effects on DNA methylation										
cg14850728	0.85 (0.04)	0.0004	7.93E−05	1.38E−06	chr11	119294941	THY1; USP2-AS1	0.85 (0.03)	2.58E−06	0.95
cg18904161	0.85 (0.02)	0.0002	4.53E−05	2.23E−06	chr6	137398100	NA	0.85 (0.03)	2.45E−05	0.45
cg08062613	0.64 (0.07)	0.0006	0.00013	3.11E−06	chr15	70275937	NA	0.54 (0.09)	−0.0002	0.04
cg01227815	0.81 (0.02)	0.0002	5.13E−05	3.65E−06	chr11	71597184	LOC100133315	0.81 (0.03)	−4.28E−05	0.18
cg15561613	0.11 (0.05)	−0.0007	0.00014	4.41E−06	chr1	245851610	KIF26B	0.13 (0.08)	9.65E−05	0.27
cg11251012	0.87 (0.02)	0.0002	4.74E−05	4.63E−06	chr1	213021561	C1orf227	0.87 (0.02)	−7.29E−06	0.75
cg02200032	0.78 (0.05)	0.0006	0.00013	8.06E−06	chr1	245270486	EFCAB2; EFCAB2	0.70 (0.12)	0.0002	0.20
cg03631474	0.23 (0.09)	−0.0009	0.00019	1.05E−05	chr6	36734511	CPNE5	0.25 (0.16)	0.0001	0.39
cg14325112	0.78 (0.03)	0.0003	7.37E−05	1.14E−05	chr9	4115070	GLIS3; GLIS3	0.73 (0.06)	5.45E−06	0.92
cg17138030	0.90 (0.02)	0.0002	5.17E−05	1.15E−05	chr11	119660621	NA	0.90 (0.03)	−1.41E−06	0.96
cg22818632	0.87 (0.03)	0.0002	5.39E−05	1.77E−05	chr16	84221234	TAF1C; TAF1C; TAF1C; TAF1C; TAF1C; TAF1C; TAF1C	0.88 (0.03)	5.14E−06	0.85
cg26342342	0.85 (0.03)	0.0004	8.15E−05	2.05E−05	chr2	29437671	ALK	0.79 (0.07)	7.16E−05	0.29
cg26721644	0.49 (0.12)	0.0012	0.00027	2.29E−05	chr9	78936369	PCSK5	0.39 (0.16)	−3.58E−05	0.81
cg15460536	0.74 (0.05)	−0.0005	0.00011	2.70E−05	chr1	9938595	CTNNBIP1; CTNNBIP1	0.75 (0.07)	1.08E−05	0.89
cg04097500	0.77 (0.05)	0.0005	0.0002	3.09E−05	chr19	34161531	CHST8; CHST8	0.68 (0.10)	−6.95E−05	0.52

Selection of the top 15 CpG sites associated with each early maternal dietary score in the PEARS study from model 2 and comparison of the effects on these CpG sites in Lifeways. Effect estimates represent the difference in DNA methylation per 1-unit increase in maternal early-pregnancy GI/GL.

Abbreviations: EWAS, epigenome-wide association study; GI, glycemic index; GL, glycemic load; PEARS, Pregnancy Exercise and nutrition Research Study.

a PEARS regression fully adjusted model is presented including child sex, gestational age, maternal smoking during pregnancy, batch effect, estimated cell-type proportions, parity, maternal education, maternal age, body mass index, ethnicity, and birthweight.

b Lifeways model 2 is adjusted for child sex, batch (plate variable), maternal smoking during pregnancy, cellular composition, parity, education, maternal age, body mass index, birthweight, and gestational age.

## Discussion

In this high-risk population of pregnant women who were overweight and obese, we observed moderate associations of maternal glucose or insulin homeostasis and glycemic dietary scores with offspring cord blood DNAm.

Insulin concentrations in late pregnancy were positively associated with DNAm changes at birth on the cg12082129, which is located near transcription start sites of the *RNF214* and *PCSK7* genes. Maternal indicators of insulin resistance and β-cell function in early pregnancy were associated with lower methylation at the CpG site cg03158092 located near the *SYN3* gene and the CpG site cg05985988 located near the *JARID2* gene, respectively. Inversely, maternal insulin sensitivity was associated with higher methylation at the CpG cg04976151 on the *POLR2C* gene. We caution, however, that the microarray probe for cg05985988 coincides with SNPs with minor allele frequencies >1%, so DNAm measurements may be influenced by genetic variation (43). However, we did not remove these probes that coincide with SNPs prior to analysis, as their removal would not much improve *P-*value adjustment for multiple tests and we preferred to maximize the information.

An increase in maternal GL scores at late pregnancy was positively associated with DNAm at birth at the CpG site cg11955198, upstream of the non-protein coding RNA long intergenic non-protein coding RNA 1150 (*LINC01150*) gene (44), while maternal GI changes during pregnancy were inversely associated with DNAm at 1 CpG site, cg03403995 located near the *OCA2* gene. Effects remain similar in the different models with following adjustment for a range of potential confounders. No associations with the other glucose biomarkers or GDM were observed.

We did not observe an intervention effect on newborn DNAm, and we found that associations with CpG sites in all newborns were similar in those born from mothers included in the control group. These observations suggest that the intervention might not modify DNAm signatures of other late-pregnancy exposures. Finally, we found no evidence of replication with associations identified in previous EWAS (10, 14, 15, 39). Replication of analyses in Lifeways does not show any associations with glycemic and insulinemic scores. Overall, our findings indicate that programming effects of maternal glycemic and insulinemic traits have weak and potentially complex effects on epigenetic markers.

### Interpretation

Maternal insulin concentrations at late pregnancy were associated with a CpG site located near the *RNF214* and *PCSK7* genes. *RNF214* is a protein-coding gene linked to disorders including Ehlers-Danlos syndrome, a group of heritable tissue disorders (45).The *PCSK7* gene encodes enzymes that process protein and peptide precursors trafficking. It may act as an important mediator of adipocyte differentiation and iron homeostasis and may imply changes in fasting insulin levels (46). Maternal IR was associated with a CpG site located near the *SYN3* gene, classified as a member of the synapsin gene family involved in brain functioning (47). The maternal pancreatic β-cell function association implies the *JARID2* gene, which encodes a protein with an essential role in embryonic development, including heart and liver development, neural tube fusion process, and hematopoiesis (48, 49). *JARID2* is also required for the complete activation of the insulin-producing β-cell differentiation program and the formation of the proper β-cell population in newborn mice (50). Maternal IS was associated with a CpG located in the *POLR2C* gene, a protein coding gene in the transcription of the DNA into RNA process (51).

The *LINC01150* gene, reported with maternal GL, is an RNA gene located on chromosome 11 (p15.5). Few data have been published to date; however, 1 study reported that this region of human chromosome contains a large cluster of imprinted genes that control growth and development and notably the H19 imprinted maternally expressed transcript (*H19*) gene (52). Finally, maternal GI changes were related to the *OCA2* gene, a protein coding gene that plays a role in the transport of tyrosine, the precursor of the melanin synthesis. It is involved in mammalian pigmentation and may control skin and eye color variation (53).

### Comparison With the Literature

There is limited evidence for a relationship between maternal glucose or insulin homeostasis and offspring DNAm. Maternal glucose and insulin concentrations were not associated with any of the previously reported maternal GDM or hyperglycemic CpGs evaluated in our look-up analyses, probably due to the fact that populations had different characteristics (10, 14, 15, 39). The UPBEAT RCT included only obese women with a population BMI of 36.5 kg/m² with higher levels of fasting glucose than in our study. Our study population is relatively healthier with a mean of BMI around 29.8 kg/m² and with a low percentage of women who developed GDM. A recent meta-analysis from 7 pregnancy cohorts among 3677 mother-newborn pairs (10) similarly included women with a wider BMI range than our study (BMI range: 23.5-37 kg/m²). The recent meta-analysis by Tobi et al did not reveal robust associations with maternal glucose and insulin levels. In that study, the BMI range was lower than in our study (23.9-28.8 kg/m^2^), and biomarkers were measured at different time points during pregnancy. Additionally, another study conducted in Singapore on 830 mother-offspring dyads revealed that mid-pregnancy fasting glucose and 2-hour plasma glucose are associated with unique epigenome-wide effect size profile. Average prepregnancy BMI was 22.7 kg/m^2^ and comprised women of different ethnic groups (54). Population included in Lifeways was as well a general obstetric population with lower BMI, around 23.4 kg/m². Moreover, lack of replication could be explained by differences in design, timing of sample collection, and tissue type (saliva vs cord blood). Associations reported in other previous studies may include false positives due to failure to adjust for multiple tests or for important confounding factors such as maternal BMI and cell heterogeneity (55–57). Other differences to our study potentially include differences in how GDM is diagnosed as there is no universally agreed-on set of diagnosis criteria. Some previous studies stratified by GDM treatment (58, 59); however, this information was missing in our study. The Generation R study did not observe associations of maternal early-pregnancy nonfasting glucose and insulin concentrations with offspring cord blood DNAm even with a much larger population of 935 mothers. Lastly, it is possible that the nonreplication across studies could be due to some results being false positives. Finally, our associations with GI and GL in pregnancy were not replicated in a recent meta-analysis of EWAS association with maternal dietary GI and GL (60).

Finally, there is limited evidence regarding interventional studies on maternal diet during pregnancy and DNAm. The ROLO study (Randomised cOntrol trial of LOw GI diet vs no dietary intervention to prevent recurrence of fetal macrosomia) did not report any DNAm differences between the intervention and control groups in cord blood and saliva of around 60 children at birth and 5 years old, respectively (61, 62). Recent evidence shows that combining digital interventions with motivational human interaction has increased engagement and behavior changes (17). Women with a higher BMI may perceive more barriers and have lower self-efficacy to improve their lifestyle, diet, or exercise behavior during pregnancy (63). Promoting meaningful change in these women is a complex and challenging issue that requires a multifactorial approach based on determinants of behavior information, motivation, and social and environmental context (64).

### Strengths and Limitations

The major strengths of this study are the RCT design, availability of extensive information on maternal plasma glucose and insulin concentrations, and comprehensive food diaries. Furthermore, we have used the updated EPIC array, which has increased coverage over important regulatory regions. We were able to adjust for a large number of potential confounders and for estimated cell-type proportions. We also used adjustments for multiple testing and carefully evaluated the effects of DNAm outliers to reduce the chance of identifying false positives.

However, this study was not without limitations. We used various derived measures of insulin sensitivity and β-cell function. Even though these measures correlate reasonably well with direct measures, they can be interpreted only as proxy. The relatively small sample size may have limited our statistical power to detect more significant associations and biological impacts of the intervention and may explain differences with prior findings. Caution is necessary when interpreting the results, and replication in other high-risk population will be necessary. Second, the methylation changes observed were modest, and the functional relevance of these changes is still unclear. Depending on the location of the CpG site, this may affect the expression of associated or surrounding genes with consequences for development and cell function. Further investigation would be necessary to assess the functional implication of our results. However, no gene expression data was available from the samples to determine whether the methylation changes observed were associated with variation in gene expression. Additionally, we limited our analysis to cord blood DNAm, which may miss associations in other relevant tissues such as placenta, body fat, liver, or pancreas.

## Conclusion

In summary, maternal pregnancy measures of glucose and insulin homeostasis and maternal dietary glycemic indices among women who were overweight and obese were associated with moderate changes in cord blood DNAm. We did not observe an effect of the antenatal lifestyle intervention on DNAm. Additionally, we found no evidence of replication of associations we have identified in previous EWAS. Maternal indicators of insulin levels, insulin resistance, or sensitivity were related with CpG sites located in genes whose functions include brain development and regulation of insulin processes. Maternal GL and variation in GI were associated with CpG sites located in regions of human chromosome involved in embryonic development or the determination of phenotype characteristics. However, these findings indicate complex effects, and the impact of methylation changes within these genes on subsequent health merits future investigation. Larger studies are required to fully explore the impact of interventions on childhood epigenome.

## Data Availability

Some or all of the data sets generated and/or analyzed during the current study are not publicly available but are available from the corresponding author on reasonable request.

## Abbreviations

BMI: body mass index
DM: diabetes mellitus
DNAm: DNA methylation
EWAS: epigenome-wide association study
FPI: fasting plasma insulin
FPG: fasting plasma glucose
GDM: gestational diabetes mellitus
GI: glycemic index
GL: glycemic load
HOMA-IR: homeostatic model assessment insulin resistance
II: insulinemic index
IL: insulinemic load
IR: insulin resistance
IS: insulin sensitivity
LIMIT: limiting weight gain in overweight and obese women during pregnancy to improve health outcomes
OGTT: oral glucose tolerance test
PACE: Pregnancy and Childhood Epigenetics consortium
pp: postprandial
PEARS: Pregnancy Exercise and Nutrition Research Study
QUICKI: quantitative insulin sensitivity check index
RCT: randomized controlled trial
SNPs: single-nucleotide polymorphisms
UPBEAT: UK Pregnancies Better Eating and Activity Trial
